# Case Report: Diagnosis and treatment of giant thoracodorsal liposarcoma

**DOI:** 10.3389/fonc.2025.1570796

**Published:** 2025-10-16

**Authors:** Kailin Liu, Baoyan Zhao, Ningning Ren, Peng Zhao, Zhihao Wang, Xia Li, Yuyang Li, Chong Geng

**Affiliations:** ^1^ Department of Breast and Thyroid Surgery, Shandong Provincial Hospital Affiliated to Shandong First Medical University, Jinan, Shandong, China; ^2^ Shandong Cancer Hospital and Institute, Shandong First Medical University and Shandong Academy of Medical Sciences, Jinan, Shandong, China; ^3^ Department of Breast Surgery, The Second Affiliated Hospital of Zhengzhou University, Zhengzhou, Henan, China; ^4^ Department of Ultrasonography, Shandong Provincial Hospital Affiliated to Shandong First Medical University, Jinan, Shandong, China

**Keywords:** giant liposarcoma, tumor microenvironment, macrophages, transcriptomic analysis, surgical excision

## Abstract

**Rationale:**

Liposarcoma is a prevalent malignant tumor of soft tissue; however, those in the thoracic and back regions are rare, and surgery is particularly challenging due to their complex anatomical structures.

**Case presentation:**

A 90-year-old man was admitted to the hospital due to a massive lump in the thoracodorsal region. Computerized tomography imaging revealed a large heterogeneous lesion with lipid density in the left axillary chest wall area, with a maximum cross-sectional area of approximately 17.4 × 14.2 cm. The histopathologic report showed a well-differentiated liposarcoma.

**Clinical discussion:**

Early detection and timely intervention of liposarcoma are critical, particularly for large liposarcomas in the thoracodorsal region because of the surrounding vital structures, such as the brachial plexus, axillary vein, and axillary artery. We found that the liposarcoma was infiltrated by M2 macrophages, with the central region exhibiting high rates of M0 macrophages and Treg cells. This immunosuppressive tumor microenvironment may provide a potential target for the development of immunotherapeutic strategies.

## 1 Introduction

Liposarcoma is the second most common type of soft tissue sarcoma that originates from adipose tissue (1). It is frequently found in the abdomen and limbs; liposarcomas in the thoracic and back regions are relatively rare, and surgery in these regions is particularly challenging due to their complex anatomical structures (2). The present case showed a huge liposarcoma located in the thoracic and back regions of an older adult patient and represents the first case recorded in our hospital. According to histological characteristics, liposarcoma could be classified into several subtypes (3, 4). Well-differentiated liposarcoma was the most common subtype, with low malignancy, slow growth, and a propensity for local recurrence (5). Dedifferentiated liposarcoma, derived from well-differentiated liposarcoma, exhibited higher malignancy and metastatic potential. Myxoid liposarcoma, often observed in the limbs, was associated with specific chromosomal translocations and had a relatively favorable prognosis, making it the second most common subtype (5). Round cell liposarcoma, a highly malignant variant of the myxoid type, was prone to distant metastasis (6–8). Pleomorphic liposarcoma, the subtype with the highest malignancy, had a poor prognosis.

In a well-differentiated liposarcoma, the most prominent molecular feature is gene amplification in the 12q13–15 chromosomal region, with MDM2 and CDK4 identified as the principal driver genes. This amplification is observed in approximately 90% of well-differentiated liposarcoma cases (9). Amplification of MDM2 can inhibit p53 activity, resulting in cell cycle evasion and uncontrolled proliferation, which represents one of the key mechanisms underlying tumorigenesis (10). In addition, single-cell studies have suggested that well-differentiated liposarcoma may originate from adipocyte stem cells and give rise to tumor heterogeneity through clonal evolution (3).

In this report, we describe our surgical findings and transcriptome sequencing results on the excised tissue to assess the characteristics of the core region of the tumor core, the tumor–normal tissue interface, and the adjacent normal adipose tissue to provide evidence for future clinical treatment and prognosis.

## 2 Case presentation

### 2.1 Patient information

**Figure 1:**
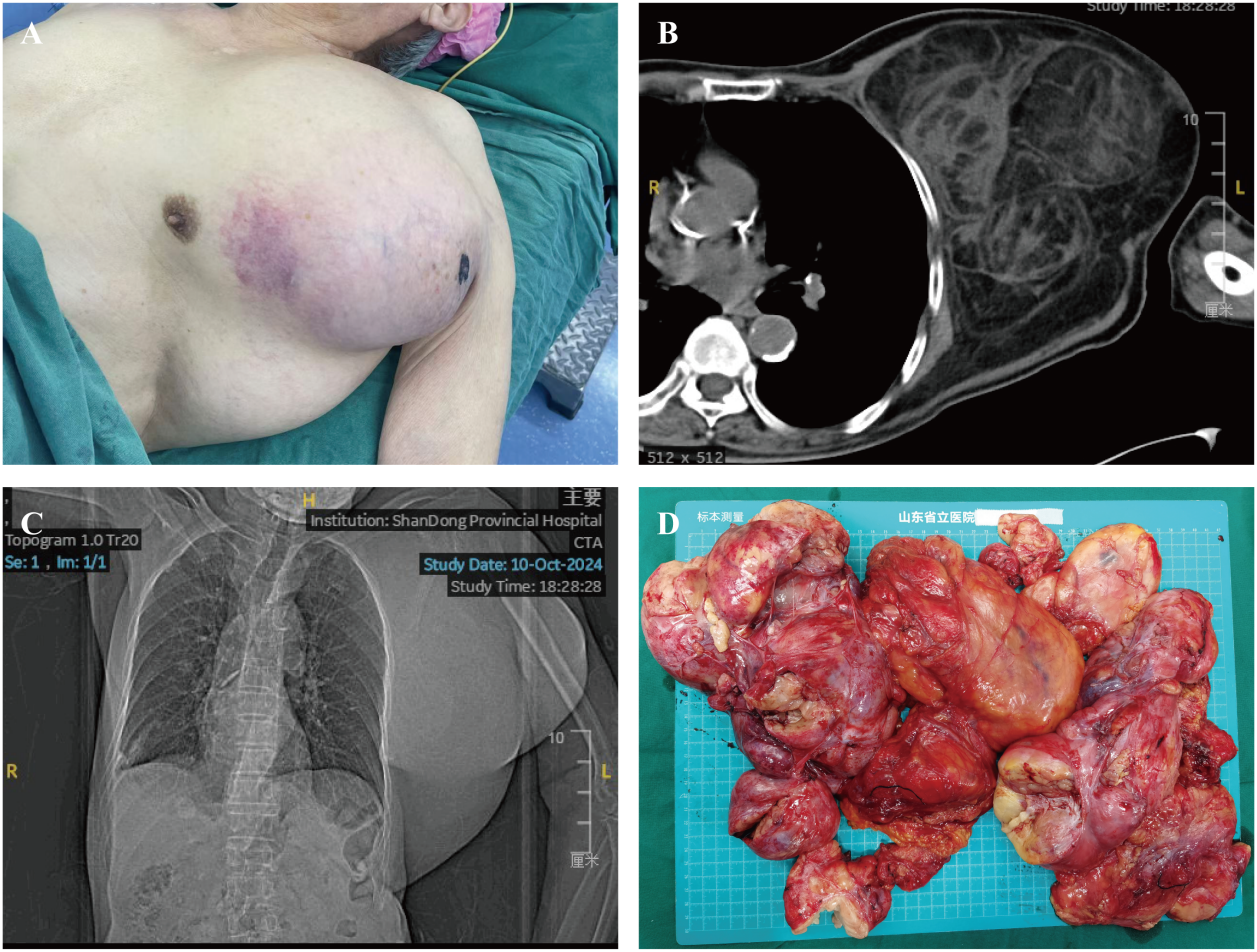
Preoperative imaging and gross specimen of the patient. **(A)** Preoperative image of the patient showing a prominent mass in the thoracic and dorsal region. **(B, C)** Chest CT revealing a thoracic-dorsal space-occupying lesion predominantly composed of fat density. **(D)** Gross specimen of a large liposarcoma.

A 90-year-old man was admitted to the Shandong Provincial Hospital due to a huge lump in the thoracodorsal region. The patient had noticed a small lump in his left armpit seven years ago. This lump was initially asymptomatic, and the patient did not receive any treatment before. However, this lump has grown rapidly over the last few years, resulting in prominent protrusions in the thoracodorsal region and causing a significant restriction in activities accompanied by numbness and loss of sensation in the left upper limb (**Figure 1A**). The patient’s family has no history about a similar tumor.

### 2.2 Clinical diagnosis

The tumor in this patient is considerably large and multi-lobed, infiltrating to parts of the left chest and back. The physical examination indicated that the texture of the tumor is rigid upon palpation and has a relatively fixed position. CT imaging showed a large lipid-rich heterogeneous lesion in the left axillary chest wall area. The shape is irregular, and the maximum cross-sectional area is approximately 17.4 × 14.2 cm (**Figures 1B, C**). Additionally, the hematological tests showed no significant finding, the patient’s alpha-fetoprotein was 1.61 ng/mL, carcinoembryonic antigen was 2.43 ng/mL, carbohydrate antigen 125 was 6.83 U/mL, and carbohydrate antigen 199 was 12.2 IU/mL.

### 2.3 Surgery intervention

Due to the advanced age of the patient, we adopted a more cautious surgical approach. A shuttle-shaped incision approximately 20 cm in length was made along the surface of the tumor, and the tumor was separated and removed layer by layer along its capsule. We found that the anatomical structures in the axillary region had been destroyed due to the infiltration of the tumor. Due to the massive size of the tumor, anatomical landmarks such as the axillary vein, cephalic vein, thoracoacromial artery, as well as the axillary vein and artery within the axilla, the circumflex scapular artery, subscapular artery, and its accompanying nerves, the thoracodorsal nerve and artery, and the long thoracic nerve and lateral thoracic artery were all compressed and displaced, making them difficult to identify, with only localized protrusion visible. To minimize bleeding and preserve as many vessels as possible to maintain postoperative muscle perfusion—thereby reducing the risk of tissue necrosis and secondary debridement—blunt dissection was performed along the tumor’s outer capsule. During the dissection, an attempt was first made to locate the axillary vein and artery. However, because the tumor severely compressed the vessels, the course of the axillary vessels could not be clearly identified; thus, all visible vessels were preserved until a distinct axillary artery pulsation was palpated, confirming its location. Using the course of the axillary artery as the main anatomical reference, surrounding vascular and neural structures were further explored. Intraoperatively, the brachial plexus, axillary vein, circumflex scapular artery, subscapular artery and its accompanying nerves, thoracodorsal nerve and artery, and long thoracic nerve and lateral thoracic artery were identified in sequence. After clearly identifying and protecting these critical structures, the tumor was divided into two parts. Following the vascular course, each relevant vessel was sequentially clamped.

The mass removed weighed approximately 4 kg, with dimensions of approximately 30 × 29 × 8 cm (**Figure 1D**). The surgery lasted approximately 3 hour. The patient was transferred to the ward for monitoring of vital signs and received symptomatic treatment during the postoperative period. The patient’s vital signs remained stable.

### 2.4 RNA isolation and library preparation

Total RNA was extracted from tumor tissues using the RNeasy mini Kit (Qiagen, Germany). Paired-end libraries were synthesized using the TruSeq^®^ RNA Sample Preparation Kit (Illumina, USA) according to the manufacturer's instructions. The libraries were sequenced on the Illumina NovaSeq 6000 platform (Illumina, USA). Raw data (raw reads) in fastq format were processed using Trimmomatic, and clean reads were mapped to the human genome (GRCh38) using HISAT2. The read counts of each gene were obtained with HTSeq-count.

### 2.5 Data pre-processing and transcriptomic analysis

The tumor purity and the presence of infiltrating stromal/immune cells in tumor tissues were estimated using ESTIMATE package (v 1.0.13) in R (v 4.3) based on the transcriptomic data. The abundances of immune cell types were predicted using CIBERSORT package (v 0.1.0).

### 2.6 Postoperative analysis

**Figure 2:**
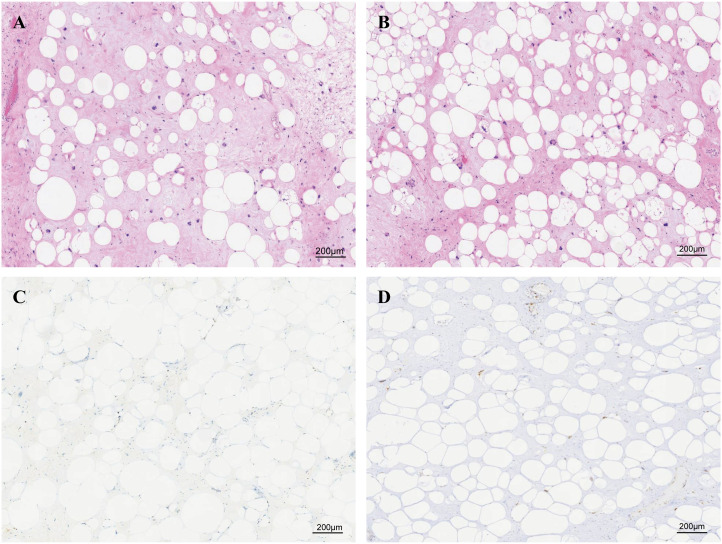
Paraffin-embedded sections stained with H&E and immunohistochemical diagnosis of liposarcoma. **(A, B)** Paraffin-embedded sections stained with H&E, consistent with the diagnosis of lipoma-like liposarcoma. Immunohistochemical staining of **(C)** Ki67 and **(D)** MDM2.

The tumor samples were formalin-fixed and paraffin-embedded (FFPE) for histochemical analysis. We confirmed that this tumor was a lipoma-like liposarcoma by its morphology (**Figures 2A, B**). The immunohistochemical analysis showed that this tumor was CDK4-positive, MDM2-positive, approximately 5% positive for Ki-67, S100-negative, and P53-negative (**Figures 2C, D**).

**Figure 3:**
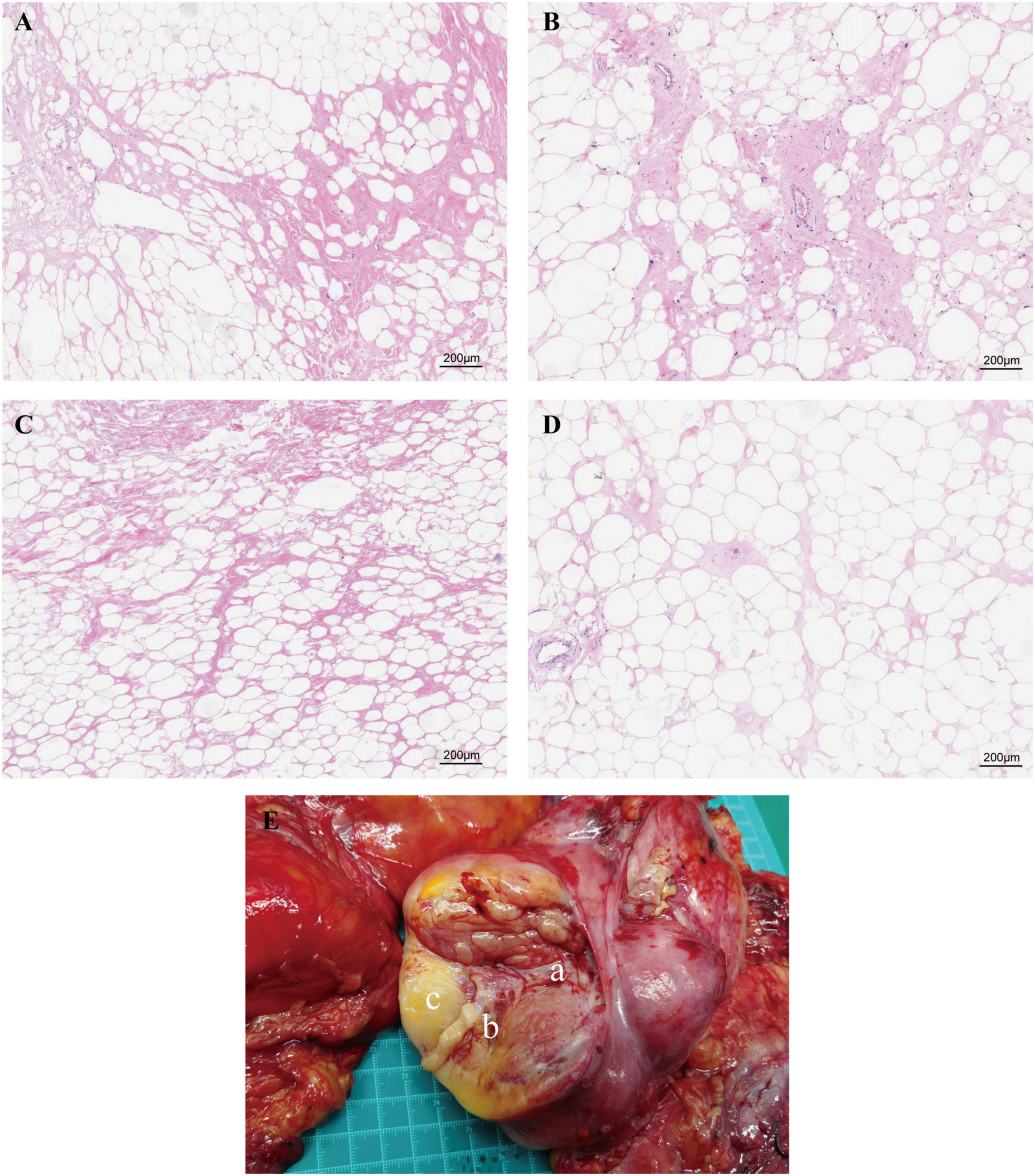
Paraffin-embedded sections of the four sampling sites stained with H&E. **(A)** The central region exhibits significant stromal fibrosis and remodeling. **(B)** The interface region shows marked immune cell infiltration. **(C)** The gelatinous region displays loose tissue architecture with no stained nuclei. **(D)** Adjacent adipose tissue demonstrates relatively normal adipocyte morphology with minimal tumor cell infiltration. **(E)** Schematic representation of tissue sampling sites. Figure part labels a, b, and c correspond to regions A (central region), B (interface region), and C (gelatinous region) of the tumor.

Additionally, given the large volume and heterogeneous texture of the tumor, we have sampled the core area of the tumor, the transition area between the tumor and normal adipose tissue, the gelatinous area, and the adjacent adipose tissue area for further analysis. The results of H&E staining (**Figure 3**) suggested that the core region showed significant stromal fibrosis and remodeling (**Figure 3A**); the interface region (**Figure 3B**) showed significant immune cell infiltration; the jelly-like region **Figure 3C** showed loose tissue structures without any stained cell nuclei; and the adjacent adipose tissue **Figure 3D** exhibited relative normal adipocyte morphology with minimal tumor cell infiltration.

**Figure 4:**
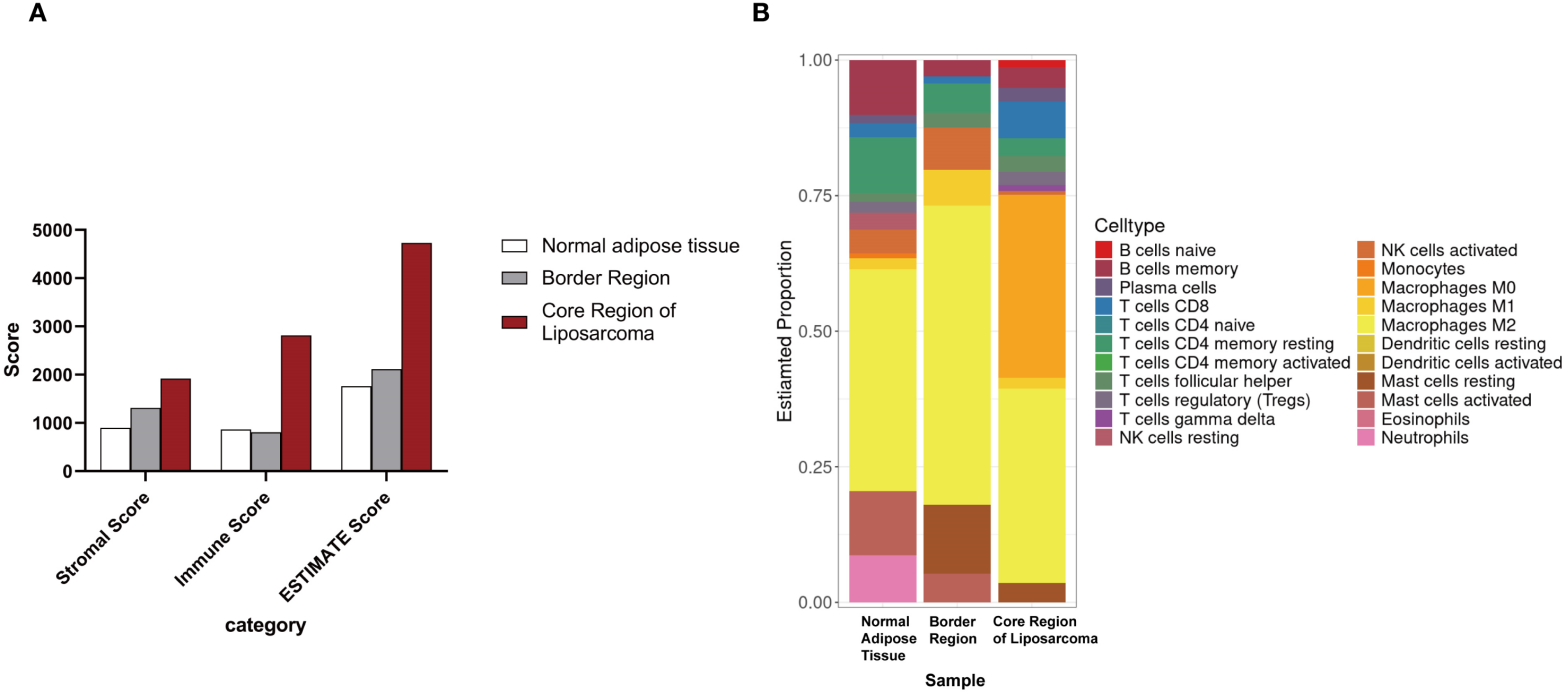
Transcriptomic analysis of tumor samples. **(A)** ESTIMATE analysis of different regions of the liposarcoma. **(B)** CIBERSORT analysis of different regions of the liposarcoma.

We also performed transcriptomic analysis on these samples. The ESTIMATE analysis showed that the core region of the liposarcoma exhibited the highest stromal and immune scores. The scores for the transition area fell between those of the tumor core and the adjacent adipose tissue (**Figure 4A**). The jelly-like region was excluded from this analysis due to insufficient RNA yield. The CIBERSORT analysis showed that while the core region was enriched with CD8+ T cells, immunosuppressive Treg cells, and non-activated macrophages, whereas, both the transition area and the adjacent adipose tissue were dominated with anti-inflammatory M_2_ cells, suggesting an immunosuppressive tumor microenvironment (TME) (**Figure 4B**).

## 3 Discussion

Liposarcoma is a highly heterogeneous malignant tumor. Radical surgery is currently the main choice for these patients. Although clinical trials related to targeted therapy and immunotherapy have demonstrated additional therapeutic options for liposarcoma, these agents are still in the experimental stage, and standardized clinical treatment protocols are lacking (11–14). Liposarcomas can occur anywhere in the body but are relatively rare in the thoracodorsal region. Early detection and timely intervention of liposarcoma are critical, particularly for large liposarcomas in the thoracodorsal region. Due to their infiltrative growth, these tumors often lead to compression or invasion of surrounding vital structures, such as the brachial plexus. In the present case, the patient reported numbness and loss of sensation in the left upper limb prior to surgery. Special attention should be paid to the complex anatomical structures of the axillary region. Complete resection while preserving the brachial plexus, axillary vein, and axillary artery is essential to minimize postoperative functional deficits and complications. Furthermore, treatment of older adult patients requires a more comprehensive evaluation of their overall health status and a personalized surgical plan.

According to histological characteristics, liposarcomas can be classified into well-differentiated, dedifferentiated, myxoid, round cell, and pleomorphic liposarcoma (3, 4). Well-differentiated liposarcoma is the most common subtype, with characterized by malignancy, slow growth, and with a five-year survival rate exceeding 70% (5). The pathogenesis of liposarcoma is complex and depends on the histological subtype. Gene alterations, such as MDM2 amplification, p53 mutations, and specific chromosomal translocation t(12;16), may contribute to the development of a liposarcoma (15). The present case was a large well-differentiated subtype with high expression levels of MDM2 according to our immunohistochemical and transcriptomic analyses. Since MDM2 is a key negative regulator of p53, its overexpression leads to p53 pathway inactivation, thereby promoting nucleotide synthesis and tumor growth (15, 16).

In addition to the clinical characteristics of the tumor, the tumor microenvironment (TME) plays a pivotal role in the progression and prognosis of liposarcoma. The TME is a complex and dynamic network comprising stromal components, immune cells, inflammatory factors, chemokines, and extracellular matrix (17, 18). In the core region of the present liposarcoma, the TME exhibits high stromal and immune scores, reflecting substantial remodeling of cancer-associated fibroblasts (CAFs) and the ECM. CAFs contribute to tumor progression by secreting collagen and matrix metalloproteinases (MMPs), which promote tumor migration, invasion, and resistance to therapy, thereby enhancing tumor aggressiveness (19). In addition, CAFs and cancer cells can transmit mechanical signals through heterotypic adhesive junctions mediated by E-cadherin/N-cadherin, inducing mechanotransduction responses in cancer cells and further driving their invasive behavior (20).

As a distinct subset of T cells, regulatory T cells (Tregs) can suppress anti-tumor immune responses (21). Tregs promote tumor cell growth and dissemination through mechanisms such as inhibiting the function of antigen-presenting cells (APCs), competitively consuming key cytokines essential for effector T cell activation and function, and secreting immunosuppressive soluble factors, thereby shaping an immunosuppressive TME (22). Such an immunosuppressive TME is often associated with poor prognosis and low response rates to immunotherapy. In this study, the proportion of Tregs in the tumor core region was significantly increased, suggesting that they may promising potential target for future immunotherapeutic strategies.

Moreover, our CIBERSORT analysis revealed a significantly increased proportion of M0 macrophages in liposarcoma tissues. Previous studies have linked immune cell infiltration patterns to sarcoma prognosis, indicating that a TME enriched in M0 and M2 macrophages correlates with lower levels of CD8^+^T cell infiltration and poor prognosis. Under different microenvironmental stimuli, M0 macrophages can differentiate into either M1 or M2 macrophages. M1 macrophages inhibit angiogenesis and induce tumor cell apoptosis by secreting pro-inflammatory factors such as TNF-α, IL-12, and reactive oxygen species (ROS), whereas M2 macrophages promote tumor progression and metastasis by secreting anti-inflammatory factors such as IL-10 and TGF-β, thereby suppressing immune surveillance, stimulating angiogenesis, and remodeling the extracellular matrix (23–25).

Although M0 macrophages are in an undifferentiated state, studies have shown that they can directly enhance tumor cell proliferation and migration. In cervical cancer (CESC), M0 macrophages promote tumor cell proliferation, migration, and invasion (25–27). Co-culture experiments further confirmed that M0 macrophages markedly enhance these malignant behaviors of HeLa cells, underscoring their critical role in modulating cancer cell behavior within the TME (26). Moreover, in hepatocellular carcinoma (HCC), M0 macrophages and their associated genes have also been shown to correlate closely with clinical features and poor prognosis (25, 27). Collectively, these findings suggest that M0 macrophages may facilitate tumor immune evasion and therapeutic resistance by remodeling the immunosuppressive TME. Notably, trabectedin can regulate the TME and suppress tumor progression by inducing the selective apoptosis of monocytes/macrophages and inhibiting the production of inflammatory mediators (14). Although the specific regulatory mechanisms of M0 macrophages in liposarcoma remain unclear, selectively targeting M0 macrophages holds promise as a novel immunotherapeutic strategy for this malignancy.

## 4 Conclusion

Here we present a case of a giant thoracodorsal liposarcoma in an older adult patient. It was diagnosed as a well-differentiated liposarcoma with high MDM2 expression. The tumor was infiltrated by M2 macrophages, with the central region exhibiting high rates of M0 macrophages and Treg cells, suggesting an immunosuppressive TME. This study highlights the key features of liposarcoma within the context of TME, providing insights for potential targeted therapeutic approaches and the development of immunotherapeutic strategies.

## Data Availability

The raw data supporting the conclusions of this article will be made available by the authors, without undue reservation.
